# Early IGF-1 Gene Therapy Prevented Oxidative Stress and Cognitive Deficits Induced by Traumatic Brain Injury

**DOI:** 10.3389/fphar.2021.672392

**Published:** 2021-06-21

**Authors:** Agustín. J. Montivero, Marisa. S. Ghersi, M. Jazmín Silvero C, Emilce Artur de la Villarmois, Johanna Catalan-Figueroa, Macarena Herrera, María Cecilia Becerra, Claudia. B. Hereñú, Mariela. F. Pérez

**Affiliations:** ^1^ Instituto de Farmacología Experimental de Córdoba (IFEC-CONICET), Departamento de Farmacología, Facultad de Ciencias Químicas, Universidad Nacional de, Córdoba, Argentina; ^2^ Instituto Multidisciplinario de Biología Vegetal (IMBIV-CONICET), Departamento de Ciencias Farmacéuticas, Facultad de Ciencias Químicas, Universidad Nacional de, Córdoba, Argentina; ^3^ Escuela de Química y Farmacia, Facultad de Medicina, Universidad Católica del Maule, Talca, Chile

**Keywords:** traumatic brain injury, neuroinflammation, secondary injury, cognitive deficits, IGF-1 gene therapy

## Abstract

Traumatic Brain Injury (TBI) remains a leading cause of morbidity and mortality in adults under 40 years old. Once primary injury occurs after TBI, neuroinflammation and oxidative stress (OS) are triggered, contributing to the development of many TBI-induced neurological deficits, and reducing the probability of critical trauma patients´ survival. Regardless the research investment on the development of anti-inflammatory and neuroprotective treatments, most pre-clinical studies have failed to report significant effects, probably because of the limited blood brain barrier permeability of no-steroidal or steroidal anti-inflammatory drugs. Lately, neurotrophic factors, such as the insulin-like growth factor 1 (IGF-1), are considered attractive therapeutic alternatives for diverse neurological pathologies, as they are neuromodulators linked to neuroprotection and anti-inflammatory effects. Considering this background, the aim of the present investigation is to test early IGF-1 gene therapy in both OS markers and cognitive deficits induced by TBI. Male Wistar rats were injected *via* Cisterna Magna with recombinant adenoviral vectors containing the IGF-1 gene cDNA 15 min post-TBI. Animals were sacrificed after 60 min, 24 h or 7 days to study the advanced oxidation protein products (AOPP) and malondialdehyde (MDA) levels, to recognize the protein oxidation damage and lipid peroxidation respectively, in the TBI neighboring brain areas. Cognitive deficits were assessed by evaluating working memory 7 days after TBI. The results reported significant increases of AOPP and MDA levels at 60 min, 24 h, and 7 days after TBI in the prefrontal cortex, motor cortex and *hippocampus*. In addition, at day 7, TBI also reduced working memory performance. Interestingly, AOPP, and MDA levels in the studied brain areas were significantly reduced after IGF-1 gene therapy that in turn prevented cognitive deficits, restoring TBI-animals working memory performance to similar values regarding control. In conclusion, early IGF-1 gene therapy could be considered a novel therapeutic approach to targeting neuroinflammation as well as to preventing some behavioral deficits related to TBI.

## Introduction

Traumatic brain injury (TBI) is the consequence of an external mechanical force applied to the cranium and its content. This event may cause temporary or permanent disabilities on TBI survivors because of its consequences at physical, cognitive or psychological levels (Niedzwecki et al., 2008; Steyerberg et al., 2008; Stocchetti and Zanier, 2016; Wolfe et al., 2018). The TBI’s severity can be classified as mild (mTBI), moderate or severe taking into account the clinical presentation of a patient’s neurologic signs and symptoms (Kay et al., 1993; Katz and Alexander, 1994; Ponsford et al., 2016). Furthermore, TBI is considered a worldwide increasingly critical health problem, according to recent studies in the United States, revealing that the number of “TBI-related emergency department visits, hospitalizations, and deaths increased by 53%” from 2006 to 2014 (Centers for Disease Contr, 2019).

Injuries related to TBI can be classified into primary and secondary. The first ones, occur at the moment of trauma and can be manifested as focal injuries (e.g. skull fractures, intracranial hematomas, lacerations, concussions, penetrating wounds), or diffuse injuries (as in diffuse axonal injury) (Alain and Wang, 2008; Katerji et al., 2019). The secondary injuries appear immediately after trauma and may last for long periods (Nortje and Menon, 2004; Pearn et al., 2017). They are frequently associated with ischemia, brain edema, and neuroinflammation, and may take place in a period from days to weeks, or even months, triggering a complex cascade of intracellular signaling, which include ATP depletion, mitochondrial dysfunction, oxidative stress (OS), and cytoskeleton damage (Montivero et al., 2021). In turn, those events induce the production of toxic and pro-inflammatory mediators, such as prostaglandins, oxidative metabolites, chemokines, and proinflammatory cytokines, which can lead to lipid peroxidation, protein oxidation, enhanced blood brain barrier (BBB) disruption and brain edema to finally induce neuronal dysfunction or death (Krishnamurthy et al., 2016; Pearn et al., 2017).

It has been described that secondary injury mechanisms, as outlined above, may contribute to the cognitive deficits observed even long-term after injury. Individuals suffering from TBI of any severity could experience a cluster of symptoms for a prolonged period of time (Kushner, 1998; Ryan and Warden, 2003; McMahon et al., 2014; Stocchetti and Zanier, 2016), commonly recognized as post-concussive syndrome (PCS) (Alexander, 1995; Ryan and Warden, 2003; Stocchetti and Zanier, 2016; Montivero et al., 2021). The PCS includes a variety of symptoms ranging from cognitive symptoms (speech changes, attention loss, dysfunction in executive function and memory, or mental slowing, among others) Kaplan et al. (2018); physical and somatic symptoms (hearing and visual disturbances, sensitivity to light or sound, pain, headaches, dizziness, nausea, fatigue, sleep disruption, and even seizures) (Lozano et al., 2015; Webster et al., 2017; Kaplan et al., 2018; Wolfe et al., 2018) and emotional/behavioral symptoms (executive dysfunction, anxiety, irritability, depression, and attention deficit) (Arciniegas et al., 2005; Bryan, 2013; Paterno et al., 2017). Regretfully, it is difficult to identify those individuals at risk for PCS with the standard clinical assessment used. Nevertheless, since the working memory is one of the cognitive functions primarily affected by TBI, its clinical evaluation, including functional magnetic resonance imaging performed within the first week of injury, may contribute to predicting the patients´ outcome (Christodoulou et al., 2001; Ricker et al., 2001; Hillary et al., 2011; Wylie et al., 2015; Montivero et al., 2021).

Unfortunately, there are no available pharmacological therapies to prevent or reverse early secondary events in order to reduce long-term disabilities described in TBI patients (Paterno et al., 2017). Several neurotrophins synthesis can be locally stimulated in the brain after TBI, among them, insulin like growth factor-1 (IGF-1) (Madathil et al., 2010; Schober et al., 2010). However, TBI-induced increments in IGF-1 and IGF-1-related signaling molecules seem to be transient and probably are not enough to provide neuroprotection or stimulate sub-acute repair or regenerative mechanisms. Early studies in TBI patients have shown a reduction in serum IGF-1 levels (Agha et al., 2004; Popovic et al., 2004; Wagner et al., 2010; Zgaljardic et al., 2011; Olivecrona et al., 2013), even after mTBI Robles et al. (2009), Wagner et al. (2010), while other investigations have reported that there were either no changes Bondanelli et al. (2002) or a long-term increase in serum IGF-1 levels after injury (Wildburger et al., 2001). These differences can be due to the diverse patient population included in those studies, as well as different trauma types, gender, injury severity, patient age, and brain regions affected (Madathil et al., 2015). Nonetheless, low IGF-1 serum levels have been implicated in the development of cognitive dysfunction Trejo et al. (2004), Koopmans et al. (2006) and were positively correlated with cognitive impairment in TBI survivors tested up to a year post injury (Madathil et al., 2015). Thus, it is plausible to consider that low plasma IGF-1 levels may contribute to the primary causes of cognitive disorders after TBI (Madathil et al., 2015). However, even though early IGF-1 administration promotes regenerative events such as neurogenesis and angiogenesis in a TBI animal model Whitaker-Lea and Valadka (2017), its therapeutic potential after TBI has not yet been assessed. It should be emphasized, that different clinical trials have shown acceptable tolerability of systemic IGF-1 therapy in moderate to severe TBI patients, as well as enhanced metabolic outcome in comparison to placebo-treated patients (Hatton et al., 1997; Madathil et al., 2015). Furthermore, systemic growth hormone administration produced increments in IGF-1 levels, inducing a tendency to improve the functional outcomes in treated patients (Dubiel et al., 2018).

Considering all the evidence aforementioned, and the fact that IGF-1 gene therapy targets brain cells *in vivo* Hereñú et al. (2009) and can decrease behavioral functional impairments in aged rats (Nishida et al., 2011; Pardo et al., 2017), the aim of the present study is to evaluate the effectiveness of early IGF-1 gene therapy in the treatment of TBI for preventing OS as well as improving cognitive deficits.

## Material and Methods

### Ethics

All procedures were carried out in accordance with the Guide for the Care and Use of Laboratory Animals as adopted and promulgated by the National Institutes of Health and the EU (Eighth Edition, 2011) and approved by the Animal Care and Use Committee, Facultad de Ciencias Químicas (Res. Dec. 1194/2017 and 2336/2019), Universidad Nacional de Córdoba. In this study, a total of 97 male Wistar rats (270–310 gr) were obtained from the Department of Pharmacology‐IFEC‐CONICET vivarium (Facultad de Ciencias Químicas, Universidad Nacional de Córdoba, Argentina) and were housed in groups of 3, in plastic boxes with metallic gridded tops, using sawdust as bedding material, in a temperature and humidity controlled conditions under a 12‐h light/dark cycle (lights on at 7 am). Food and water were freely available. Experiments were made minimizing the number of animals used and their suffering.

### Animal Model of TBI

In order to mimic human diffuse TBI caused by falls or motor vehicle accidents, we used the Marmarou’s impact acceleration model (Marmarou´s IAM) that recreates global impact on the brain (Foda and Marmarou, 1994; Marmarou et al., 1994; Silva et al., 2011; Xiong et al., 2013). For this purpose, animals were anesthetized (Ketamine 55 mg/kg/xylazine 11 mg/kg) and subjected to a controlled TBI, described elsewhere (Marmarou et al., 1994; Silva et al., 2011). Briefly, the “trauma device” consists of 1 m removable aluminum tube, attached to a wooden platform, which allows a controlled impact when dropping a 45 gr brass sphere onto a stainless steel disc located above the animal head, causing a 0.45J impact when it is dropped from the mentioned height. Under anesthesia, animals were placed on the platform covered with a foam bed, with their heads located under an extreme of the tube, approximately midline between Lambda and Bregma. The stainless steel disc was located between the animal’s head and the tube, in order to prevent skull fractures. Then, they were subjected to the impact by dropping the sphere from the aluminum tube top. In the control group (Sham) the animals were anesthetized and placed on the trauma device platform, but they did not received impact.

**FIGURE 1:**
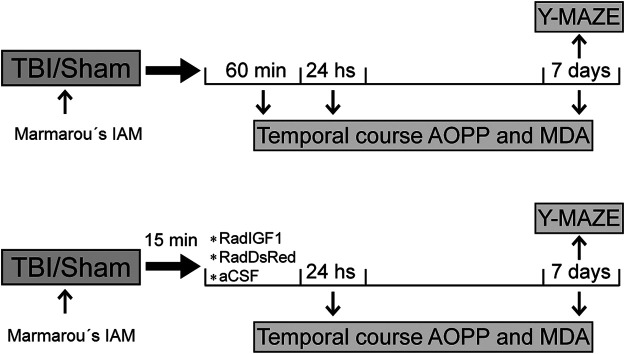
Experimental design. Chart illustrating our experimental design including Marmarou’s impact acceleration model (Marmarou´s IAM), viral vectors administration, temporal course of protein (AOPP) and lipids (MDA) peroxidation, and neurobehavioral assesements (Y-Maze test).

Once animals received the impact (TBI group) or its mimic (Sham group) they were divided into groups to perform the experiments as it was shown in Figure 1: Temporal course of OS and behavior after TBI and the effects of early IGF-1 gene therapy in OS and behavior.

### Temporal Course of Advanced Oxidation Protein Products and Malondialdehyde Quantification after TBI

Sixteen animals were subjected to the protocol described in 2.2 (TBI or Sham) and divided into four groups (Sham; TBI 60 min; TBI 24 h, and TBI 7 days) in order to evaluate AOPP and MDA concentrations at different time points after TBI (60 min, 24 h, and 7 days). After the times indicated, animals were sacrificed by guillotine, their brains were removed and Prefrontal Cortex (PFC), Motor Cortex (MC) and *Hippocampus* (HIP) were dissected.

Tissue samples were then homogenized with phosphate buffer saline (PBS) 0.1 M and centrifugated for 10 min at 4°C and 12,000g. The supernatant was diluted 1/30 for HIP and 1/20 for PFC and MC in PBS 0.1 M. AOPP and MDA determinations were made as it was previously described with modifications (Occhieppo et al., 2020). Briefly, AOPP determinations were performed accordingly to Katerji et al. (2019) and 200 µL of the diluted sample, chloramine T (0–100 μmol/L) for calibration or PBS 0.1 M pH 7 as blank, were applied in different microtiter plate wells. Then, 10 µL of 1.16 M potassium iodide and 20 µL of acetic acid (glacial) were added to each well and absorbance was immediately read at 340 nm. The AOPP concentration was expressed in chloramine units (µmol/L) per milligram of proteins.

For MDA determinations, 250 µL of trichloroacetic acid and 250 µL of thiobarbituric acid were added to 500 µL of diluted sample. Immediately after, samples were kept in boiling water for 10 min. Then, they were centrifugated at 1,000 rpm for 10 min, and after cooling to clear the supernatant from denaturalized proteins, absorbance was measured at 532 nm. Thiobarbituric acid-reactive substances were quantified using an extinction coefficient of 1.56 × 105 M^−1^cm^−1^ and expressed as nanomole of malondialdehyde (MDA) per milligram of proteins. In both AOPP and MDA determinations, tissue proteins were determined by using the Bradford reagent (Occhieppo et al., 2020).

### IGF-1 Gene Therapy after TBI

#### Viral Vectors

For animal treatment we employed recombinant adenoviral vectors (RAd) previously constructed as carriers to deliver either the therapeutic cDNA of IGF-1 gene (RadIGF1) or the red fluorescent protein from Discosoma sp DsRed (RadDsRed), as control virus (Hereñú et al., 2007). The viral dose [10^10^ plaque forming units (pfu) of the appropriate vector] was suspended in 30 µL artificial cerebrospinal fluid (aCSF- in mM: NaCl 124, KCl 2.5, NaHCO_3_ 26, MgCl_2_ 2, CaCl_2_ 2, and glucose 10; pH 7.4; 310 mosM/Kg) and administered directly into the subarachnoid space *via* Cisterna Magna. Once administered, adenoviral vectors were distributed by the cerebrospinal fluid and the ependymal cells were infected. The expression of DsRed was verified in transversal brain slices from animals subjected to TBI that received RadDsRed (see protocol below). DsRed fluorescence was more abundant in posterior brain slices near the injection site (Figure 4A) rather than anterior brain slices (Figures 4B,C).

#### Experimental Protocol

##### Effect of IGF-1 Gene Therapy in AOPP and MDA after TBI

In order to test effectiveness of CNS IGF-1 over-expression on OS markers reversion, 32 animals were randomly assigned to either Sham or TBI, which received a single administration (30 µL) of aCSF or viral suspension respectively. The procedure was carried out as previously described (Nishida et al., 2020). Briefly, 15 min after the TBI protocol, and still under anesthesia, animals’ heads were shaved and cleaned with 70% ethanol in order to expose the area of injection (Cisterna Magna). They were then placed on a stereotactic apparatus in hyperflexion prone position, in order to expand the Cisterna Magna. A 29G needle fitted to a 1 ml syringe was used to deliver viral administration. Entry into the Cisterna Magna was verified by drawing out 30 µL clear cerebrospinal fluid, which was replaced by 30 µL of viral suspension or aCSF. For the 24 h time point, TBI animals received either RadIGF1 suspension (TBI RadIGF1 group) or aCSF (TBI group); while for the 7 days time point TBI animals received RadIGF1 suspension (TBI RadIGF1 group), RadDsRed suspension (TBI RadDsRed group) or aCSF (TBI group). Sham groups were simultaneously tested at both time points, but they received 30 µL of aCSF without receiving TBI. After treatment, animals were kept undisturbed in their homeboxes until their sacrifice.

##### Effect of IGF-1 Gene Therapy in Working Memory after TBI

In order to test the efficacy of IGF-1 gene therapy in reversing cognitive deficits induced by TBI, forty-nine animals were randomly assigned to Sham or TBI. Treatment was performed as indicated in the previous paragraph, for the 7 days time point. Then, animals were placed back in their homeboxes and after 7 days they were tested in the Y-Maze task as it is described below. We obtained four groups depending on treatment: Sham, TBI; TBI RadDsRed, and TBI RadIGF1.

### Y-Maze Test

The Y-maze task allows the assessment of working memory by measuring the number of spontaneous alternations. This can be assessed by allowing mice to freely explore all the three arms of the maze. This behavior is driven by the innate curiosity of rodents to explore previously unvisited areas. The apparatus is made of three arms (50 cm long, 10 cm wide, and 20 cm high) of water-proof wood, elevated to a height of 50 cm above the floor. The test was conducted as it was described elsewhere with modifications (Xiong et al., 2013). Briefly, rats were placed at the end of arm #1 facing the center and they could choose between arm #2 and arm #3. Entry into an arm was defined as placement of the four paws into the arm and an alternation was defined as triplet of consecutive entries to different arms. The test lasted 8 min. Animals that did not reach the inclusion criteria (at least five entries and/or 2 min without a new movement or entry) were excluded. The number of total entries was also quantified as an indicator of locomotor activity.

### Statistical Analyses

Results of positive control (TBI) and experimental animals (TBI + treatment) were normalized to negative controls (Sham). The delta method was used for dispersion graphic display Polli et al. (1997), since it allows weighing how much the dispersion of each set of variables affects their means ratio dispersion. If normal data distribution was confirmed by Shapiro-Wilk test, the Student’s t-test was used when two independent groups were compared. For more than two group comparisons, data were analyzed by one-way ANOVA, followed by Tukey’s Honest Significant Difference test. On the other hand, data with non-normal distribution were analyzed by using Mann-Whitney test, or Kruskal-Wallis followed by Nemenyi test. A 95% confidence level was considered for all analyses.

For OS markers determinations and behavior performed 7 days after TBI, experiments were designed as independent groups and analyzed by using one-way ANOVA in order to reduce the number of animals used. It can be noted that the group administered with the control virus (RadDsRed) was only included in this time point or the same reasons.

## Results

### TBI-Induced Increments in Oxidative Stress Markers Last up to 7 days Post Trauma

**FIGURE 2:**
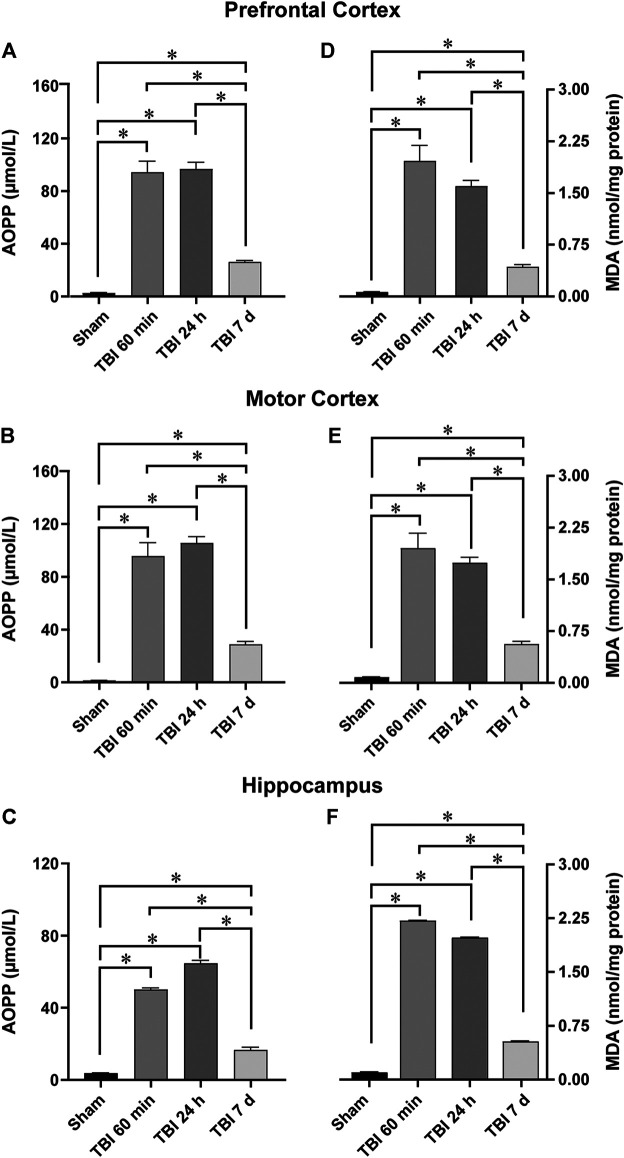
TBI induces a persistent increment in OS biomarkers. The graphs show the AOPP and MDA quantification as measurements of lipid and protein peroxidation, respectively, in brain areas neighboring the trauma area, such as **(A)** and **(D)** Prefrontal cortex; **(B)** and **(E)** Motor cortex; and **(C)** and **(F)** *Hippocampus*. Bars represent the mean ± SEM; *n* = 4. *Indicates significant difference (*p* < 0.05).

It has been recently shown that controlled cortical impact (another animal model of TBI) induced early and significant increases in lipid ROS and MDA levels, which returned to baseline seven days after TBI (Xie et al., 2019). In our model we observed a similar pattern of AOPP and MDA increments; nevertheless, even though we observed a decrease in OS levels after 7 days of TBI, they did not return to baseline. Figures 2A–C shows that the AOPP concentration in trauma adjacent regions or immediately below was significantly elevated at 60 min, reaching a maximum 24 h after TBI, when compared to Sham condition. Main effects were observed in PFC [F (3, 10) = 168.6; *p* < 0.0001] (Figure 2A), MC (Figure 2B) [F (3, 10) = 176.9; *p* < 0.0001], and HIP (Figure 2C) [F (3, 10) = 492.9; *p* < 0.0001]. The post-hoc test indicated that all groups of animals submitted to TBI were significantly different from Sham group (*p* < 0,05). Furthermore, the 7 days group was also significantly different from 60 min to 24 h groups (*p* < 0.05), indicating that there is a trend to reduce AOPP levels on day 7 after TBI, even though they are still increased when compared to Sham group in all the brain structures studied.

In the case of MDA levels, the same AOPP pattern of increment/reduction was observed. Figures 2D–F shows the inverted U-shaped pattern in MDA concentrations with main effect in PFC (Figure 2D) [F (3, 10) = 132.6; *p* < 0.0001], MC (Figure 2E) [F (3, 10) = 148.9; *p* < 0.0001] and HIP (Figure 2F) [F (3, 10) = 23,278; *p* < 0.0001]. Tukey’s post-hoc test indicated that groups from all time points were significantly higher than the Sham group (*p* < 0,05), and the 7 days group was significantly lower than 60 min and 24 h groups in all brain structures investigated (*p* < 0.05).

### Early IGF-1 Gene Therapy Prevented Increments in Oxidative Stress Markers at 24 h and 7 days Induced by TBI

**FIGURE 3:**
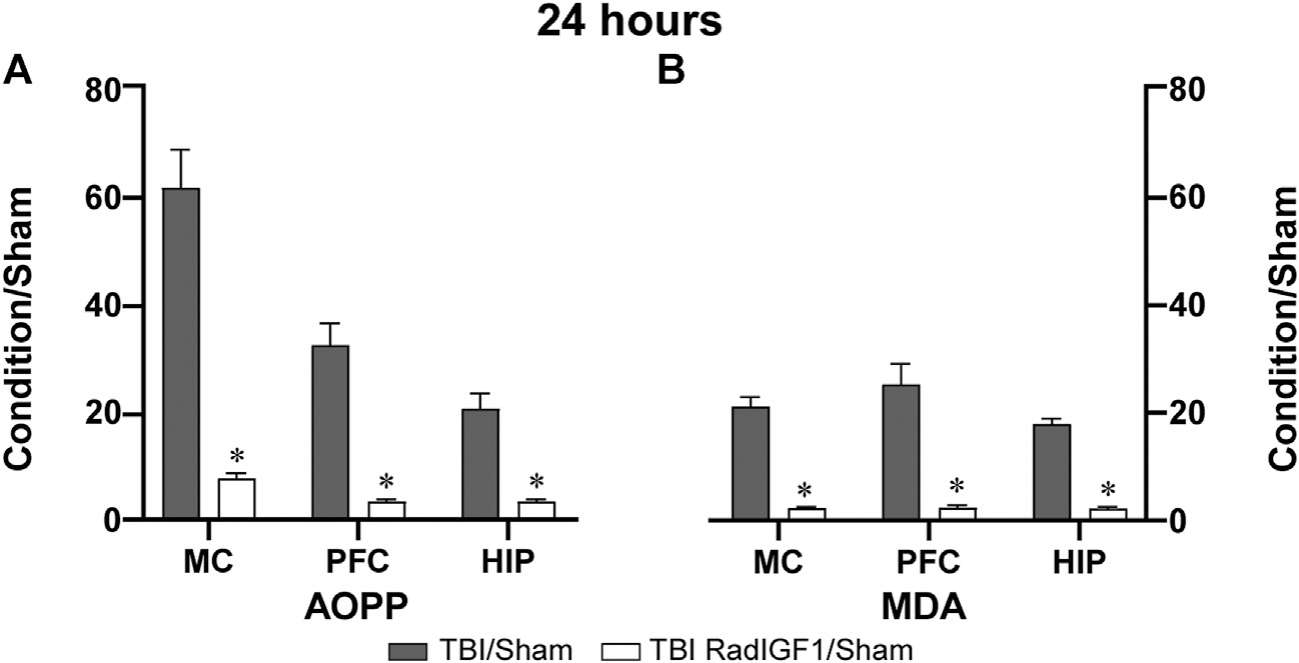
Early IGF-1 treatment reduced TBI- induced OS biomarkers 24 h after injury. The graph shows the AOPP **(A)** and MDA **(B)** quantification as measurements of protein and lipid peroxidation, respectively, in brain areas neighboring the trauma area, such as Motor cortex (MC), Prefrontal cortex (PFC), and hippocampus (HIP). Results of positive control (TBI) and treated animals (TBI RadIGF1), referred as “condition” in the *Y* axes, were normalized to negative controls (Sham). Bars represent the mean ± SEM; *n* = 4–6. *Different from TBI/Sham in each brain structure (*p* < 0.05).

24 h after TBI, AOPP concentrations in different brain structures were determined in animals that received gene therapy 15 min after the head trauma (Figure 3A). This treatment significantly reduced the AOPP mean ratios of treated condition (TBI RadIGF1/Sham) when compared with the “untreated condition” (TBI/Sham) in MC (t = 69.92; *p* < 0.000000001), HIP (t = 36.29; *p* < 0.0001) and PFC (U = 0; *p* < 0.05). Over expression of IGF-1, also impacted on MDA levels measured 24 h after TBI (Figure 3B). The mean ratios of the “treated condition” (TBI RadIGF1/Sham) were significantly reduced when compared to no treatment condition (TBI/Sham) in MC (t = 57.2; *p* < 0.00001), PFC (t = 85.35; *p* < 0.000000001), and HIP (t = 58.54; *p* < 0.000000001). These results indicate that early gene therapy prevented the peak in the AOPP and MDA levels observed 24 h after TBI.

**FIGURE 4:**
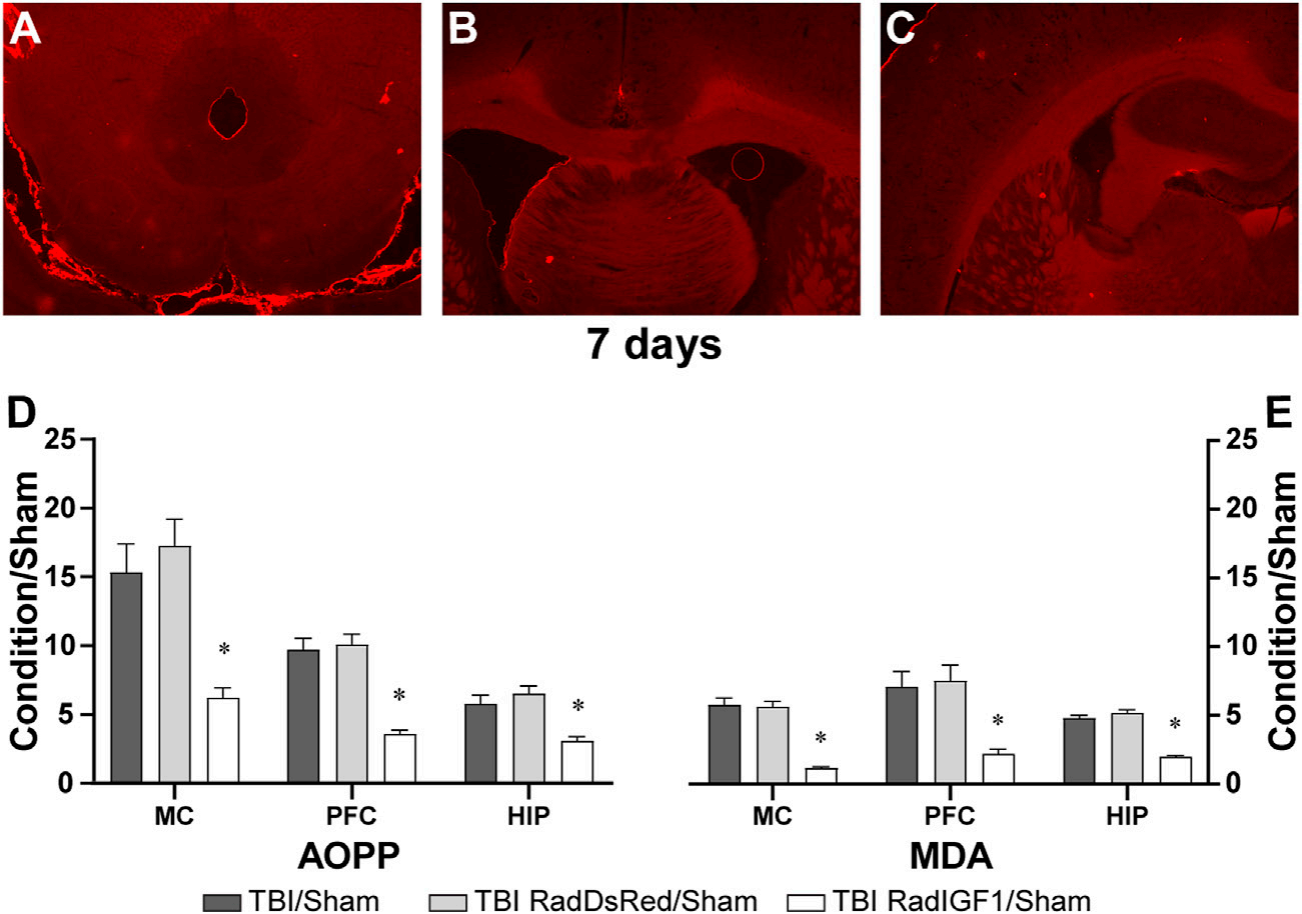
Early IGF-1 treatment prevented TBI- induced OS biomarkers 7 days after injury. Microscopic photographs of brain areas showing expression of fluorescent DsRed Protein. DsRed fluorescence is more abundant in posterior brain slices, near the injection site **(A)** rather than anterior brain slices **(B–C)**. The graph shows the AOPP **(D)** and MDA **(E)** quantification as measurements of protein and lipid peroxidation, respectively, in brain areas neighboring the trauma area, such as Motor cortex (MC), Prefrontal cortex (PFC), and hippocampus (HIP). Results of positive control (TBI) and treated animals (TBI + treatment), referred as “condition” in the *Y* axes, were normalized to negative controls (Sham). Bars represent the mean ± SEM; *n* = 4–6. *Different from TBI/Sham and TBI RadDsRed/Sham in each brain structure (*p* < 0.05).

In other groups of animals, the effect of early gene therapy 7 days after TBI over AOPP and MDA concentrations was studied (Figure 4). The following treatments were applied to animals submitted to TBI: i) control group: viral vectors containing cDNA of red fluorescent protein from Discosoma sp DsRed (RadDsRed); and ii) experimental group: viral vectors containing the therapeutic cDNA of IGF-1 gene (RadIGF1) as treatment. A main effect was observed in AOPP mean ratios of different conditions in MC [F (2, 11) = 54.98; *p* < 0.00001], PFC [F (2, 11) = 179.6; *p* < 0.0000001] and HIP [F (2, 11) = 44.26; *p* < 0.0001] (Figure 4D). In all brain structures, TBI/Sham group and control group (TBI RadDsRed/Sham group) means ratio were elevated 50% or more in comparison to the experimental group’s means ratio (TBI RadIGF1/Sham group), the post-hoc test indicated that AOPP mean ratios of experimental group were significantly different from AOPP mean ratios of untreated conditions (TBI/Sham) and from AOPP mean ratios of control treatment conditions (*p* < 0.05). Furthermore, no differences were found between AOPP mean rations of TBI/Sham and TBI RadDsRed/Sham groups. These results indicate that early gene therapy was able to prevent the sustained increment in AOPP concentrations observed up to 7 days after TBI, and this effect was due to IGF-1 over-expression and not because of viral vectors administration.

Figure 4B shows significant main effects in MDA mean ratios 7 days after TBI and treatments in MC [F (2, 11) = 133.34; *p* < 0.000001], PFC [F (2, 11) = 1468.37; *p* < 0.000000001] and HIP [F (2, 11) = 607.17; *p* < 0.00000001]. The post-hoc test in all brain structures indicated that MDA means ratio of TBI RadIGF1/Sham were significantly different regarding TBI/Sham group and TBIRadDsRed/Sham groups. Furthermore, no differences were found between MDA mean rations of both TBI/Sham and TBI RadDsRed/Sham groups. As it was described before, these results indicated that early IGF-1 over-expression after TBI was able to prevent the sustained increase observed in lipids peroxidation as a consequence of trauma.

### Early IGF-1 Gene Therapy Prevented Cognitive Deficits Observed 7 days after TBI

**FIGURE 5:**
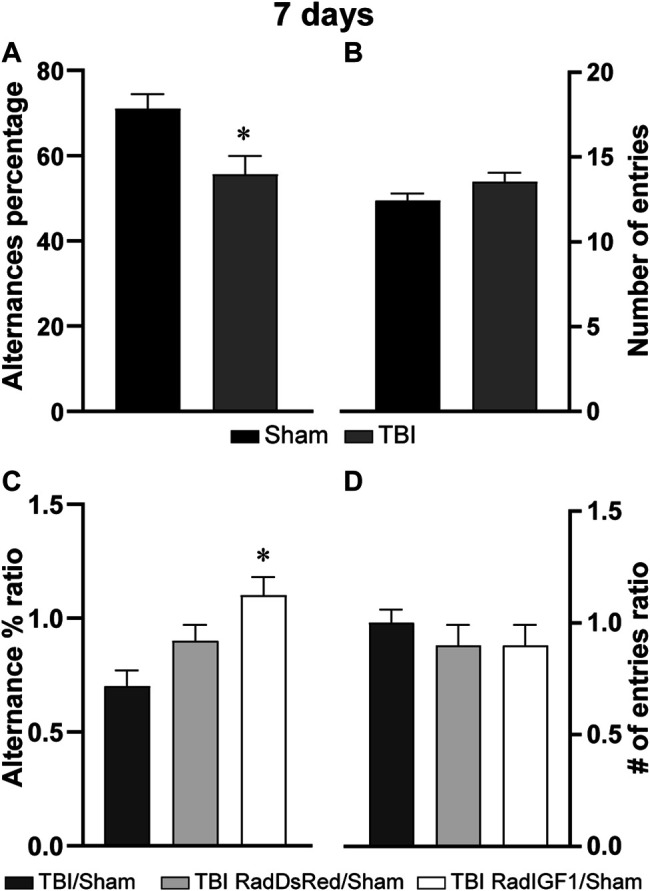
Early IGF-1 treatment prevented TBI-induced working memory deficit observed 7 days after injury. The graphs show working memory performance assessed by the Y-maze paradigm. Graphs **(A)** and **(B)** show the percentage of spontaneous alternations and percentage of spontaneous alternations in a Y-maze arena, respectively, in Sham and TBI groups. Graphs **(C)** and **(D)** represent the percentage of spontaneous alternations or number of entries ratio respectively, between TBI or TBI + treatment groups and Sham group (TBI/Sham, TBI RadDsRed/Sham, and TBI RadIGF1/Sham). Bars represent the mean ± SEM, *n* = 10–11. *Different from TBI/Sham (*p* = 0,0095).

As it was described before, working memory is one of the cognitive domains primarily affected by TBI that could predict patient’s outcome (Ricker et al., 2001; Wylie et al., 2015; Montivero et al., 2021). In preclinical studies, an individual with normal working memory will remember the arms of the Y-maze that it has already visited and will show a tendency to enter the less recently visited arm. The correct performance requires interaction across several different regions of the brain, such as the HP and PFC (Kraeuter et al., 2019). In the present work we observed, under our experimental conditions, a deficit in this domain in the TBI group (Figure 5A) since a significant reduction was observed in spontaneous alterations in the TBI group compared to Sham (t = 2,825; *p* = 0,0083). No significant differences were detected in the number of arm entries (t = 1,623; *p* = 0,1147) (Figure 5B), indicating that the reduction in spontaneous alterations was not consequence of a reduced locomotor activity.

In this experiment, the following treatments were applied to animals submitted to TBI: i) control group: viral vectors containing cDNA of red fluorescent protein from Discosoma sp DsRed (RadDsRed); and ii) experimental group: viral vectors containing the therapeutic cDNA of IGF-1 gene (RadIGF1) as treatment. The percentage of alternances and number of entries were expressed as ratios between treatments and Sham (Figures 5C,D respectively). A significant main effect in the mean ratio of the percentage of spontaneous alterations was observed between TBI/Sham, TBI RadDsRed/Sham and TBI RadIGF1/Sham groups [F (2, 31) = 5,051; *p* = 0,0131] (Figure 5C). The post-hoc test indicated that mean ratio of TBI RadIGF1/Sham group was significantly higher compared to the TBI/Sham group mean ratio. If we look at the TBI RadIGF-1/Sham group mean in Figure 5C, we can observe that it is close to 1, because spontaneous alterations of experimental group were similar to Sham, indicating a prevention of the deficits observed in the TBI in the animals that received the early IGF-1 gene therapy (Figure 5C). Furthermore, Tukey’s test showed no significant differences between TBI/Sham and TBI RadDsRed/Sham groups, showing that the control treatment did not have impact on spontaneous alternations in the Y-maze test (Figure 5C). Once again, locomotor activity was not affected by treatments, since no main effect in the number of arm entries mean ratio was found between groups [F (3, 46) = 1.649; *p* = 0,1911] (Figure 5D).

## Discussion

In this work, we showed that mTBI increases some OS markers as a result of secondary injury. These markers were observed early after TBI (60 min), reaching their highest levels at 24 h in all brain structures analyzed. In addition, even though AOPP and MDA products were significantly reduced on day 7 after TBI, they did not return to basal levels. These results are in line with studies that point out neuroinflammation as a key player in brain damage severity and patients' outcome after any kind of TBI. For instance, recognized neuroinflammation serum protein markers, such as Glial Fibrillary Acidic Protein (GFAP) and S100B, have demonstrated to have a correlation to both severity and outcome in TBI (Pelinka et al., 2004; Luoto et al., 2017). Furthermore, the more severe the TBI is, the less successful outcome is observed in patients and animal models. Many severe TBI models have shown important brain damage and correlate neuroinflammation with memory disorders (Farbood et al., 2015; Zhang et al., 2017). Thus, with our model, we have shown that even mTBI can lead to memory impairment as a sign of PCS. The observed deficits in working memory 7 days after TBI, related to impaired cognitive function, were probably the result of the early neuroinflammatory cascade triggered by secondary injury mechanisms. Working memory performance depends on the PFC integrity (Riggall and Postle, 2012; Lara and Wallis, 2015). Interestingly, we have observed an increase of OS markers in this brain structure that remains elevated over a 7-days period after TBI, compared to Sham animals. This could be explained as the result of microglia hyperactivation, as it occurs in the pathogenesis of many CNS diseases Pekny and Pekna (2016), and/or changes in cerebral blood perfusion (Babior, 2000; Block et al., 2006; Block and Hong, 2007), which in turn may affect neuronal excitability that finally shape the disrupted working memory performance observed.

Although increasing knowledge about injury mechanisms of TBI has led to a better understanding of its complex pathogenesis, pharmacological strategies focused on ameliorating them are yet missing. To date in clinical practice, medical management of TBI is tailored according to the severity of each case considering the Glasgow scale. In moderate to severe cases the treatment aim is to keep a normal intracranial pressure (ICP) in order to maintain an adequate cerebral blood pressure (CCP), which is an indicator of brain perfusion. The pharmacological armamentarium consists in sedation, neuromuscular blocking, and hyperosmolar agents that contribute to decreasing ICP (Carney et al., 2017). On the other hand, mTBI has a different approach because most of the time the ICP remains within normal ranges, thus the treatment is mainly focused on headaches management and the control of PCS- related symptoms. In these cases, painkillers are the most used medication with only evidence of improving pain, but not decreasing the symptoms duration nor improving cognitive deficits. TBI-related mood disorders can be improved by using antidepressants and psychostimulants, and yet not dealing adequately with memory deficits. Unfortunately, none of them can interfere with the neuroinflammatory cascade that could be the main cause of the sequelae after severe or mTBI (Whyte et al., 2002; Comper et al., 2005; Wheaton et al., 2011; Huang et al., 2016). In the present study we propose an early IGF-1 gene therapy, considering previous reports regarding its neuroprotective and anti-inflammatory effects (Zheng et al., 2000; Carlson and Saatman, 2018; Serhan et al., 2019). In fact, a small clinical trial showed improvement in neurological outcome in severe TBI patients, when intravenous IGF-1 was administered for several days, without effect in those with mTBI (Hatton et al., 1997). Our results indicate that locally administered IGF-1 gene therapy a few minutes after TBI, significantly reduces recognized OS markers, such as the protein and lipid (AOPP and MDA concentrations respectively) peroxidation increments observed at 24 h and impairs its long-term maintenance, also by reducing their levels 7 days after TBI. It is worth noting that this effect is specifically due to IGF-1 over-expression and not only by viruses’ administration since control viruses (RadDsRed) did not reduce AOPP nor MDA levels 7 days after TBI. With this experimental approach, we were able to express the Red protein in brain areas neighboring to the virus administration site (see Figure 4A), and since the effects were observed in frontal brain areas such as PFC and MC, the newly produced IGF-1 could be actively transported through the choroid plexus and translocated to the cerebrospinal fluid to finally reach different areas of the nervous tissue, as it was recently reported (Nishida et al., 2020). Nevertheless, further studies need to be performed to reveal changes in other players involved in the OS balance: antioxidants enzymes such as superoxide dismutase, catalase, and glutathione peroxidase, as well as accumulation of oxidation products of the proteins and lipids, like protein carbonyl and 4-hydroxynonenal (Impellizzeri et al., 2019; Siracusa et al., 2020).

The PFC plays an important role in execution of behavioral tasks that require spatial working memory, and a direct PFC-HIP pathway allows the encoding of salient spatial signals during task execution (Deakin et al., 2012). In fact, lesion- or drug-induced disturbances in HIP function certainly affect spatial working memory performance (Dillon et al., 2008; Tian et al., 2017; Ayabe et al., 2019). In the present study, we found persistent increases in OS markers in these two relevant brain structures that may affect the PFC-HIP bidirectional control required to observe spontaneous alternations. Early IGF-1 gene therapy was able to prevent the behavioral deficits observed in TBI rats after 7 days of injury. Only the group treated with vectors carrying the cDNA of IGF-1 gene showed a percentage of spontaneous arm alternations similar to the control group (Sham), while the TBI or TBI expressing Red protein groups did not show differences from each other and had lower performance rate than the controls in Y-maze spontaneous alternations. All together, these results could indicate that normal working memory can be preserved long-term after injury if critical initial secondary injury mechanisms were prevented in brain structures critical to this cognitive function. This novel therapeutic approach could be beneficial from IGF-1 peripheral administration for many reasons: first, local over-expression can be maintained even after a month from a single administration (Falomir-Lockhart et al., 2019); second, local over-expression may reduce systemic effects compared to peripheral administrations which may need massive doses in order to reach therapeutic CNS IGF-1 concentrations and lastly no doubts about treatment patient’s adherence, since administration must be performed during hospitalization, in a temporal window that guarantees the pharmacological effect.

In conclusion, results presented in this work indicate that brain focalized IGF-1 over-expression could be an effective therapeutic approach targeting neuroinflammation and also improving the cognitive deficits observed after TBI.

## Data Availability

The original contributions presented in the study are included in the article/Supplementary Material, further inquiries can be directed to the corresponding author.
